# Pathways to research impact in primary healthcare: What do Australian primary healthcare researchers believe works best to facilitate the use of their research findings?

**DOI:** 10.1186/s12961-017-0179-7

**Published:** 2017-03-02

**Authors:** Richard L. Reed, Ellen McIntyre, Eleanor Jackson-Bowers, Libby Kalucy

**Affiliations:** 1 Discipline of General Practice, Flinders University, Bedford Park, Adelaide, Australia; 2 Primary Health Care Research and Information Service, Discipline of General Practice, Flinders University, Bedford Park, Adelaide, Australia

**Keywords:** Research impact, Pathways, Primary healthcare, Policy, Clinical practice

## Abstract

**Background:**

Primary healthcare researchers are under increasing pressure to demonstrate measurable and lasting improvement in clinical practice and healthcare policy as a result of their work. It is therefore important to understand the effectiveness of the research dissemination strategies used. The aim of this paper is to describe the pathways for research impact that have been achieved across several government-funded primary healthcare projects, and the effectiveness of these methods as perceived by their Chief Investigators.

**Methods:**

The project used an online survey to collect information about government-funded primary healthcare research projects. Chief Investigators were asked how they disseminated their findings and how this achieved impact in policy and practice. They were also asked to express their beliefs regarding the most effective means of achieving research impact and describe how this occurred.

**Results:**

Chief Investigators of 17 projects indicated that a number of dissemination strategies were used but that professional networks were the most effective means of promoting uptake of their research findings. Utilisation of research findings for clinical practice was most likely to occur in organisations or among individual practitioners who were most closely associated with the research team, or when research findings were included in educational programmes involving clinical practice. Uptake of both policy- and practice-related research was deemed most successful if intermediary organisations such as formal professional networks were engaged in the research. Successful primary healthcare researchers had developed critical relationships with intermediary organisations within primary healthcare before the initiation of the research and had also involved them in the design. The scale of research impact was influenced by the current policy environment, the type and significance of the results, and the endorsement (or lack thereof) of professional bodies.

**Conclusions:**

Chief Investigators believed that networks were the most effective means of research dissemination. Researchers who were embedded in professional, clinical or policy-focussed intermediary organisations, or had developed partnerships with clinical services, which had a vested interest in the research findings, were more able to describe a direct impact of their research. This suggests that development of these relationships and engagement of these stakeholders by primary healthcare researchers is a vital step for optimal research utilisation in the primary healthcare setting.

## Background

Primary healthcare (PHC) is increasingly regarded as a critical element in improving population and individual health as well as controlling health costs [1]. A strong and robust evidence-base is required to support more effective government policy surrounding PHC systems and improved clinical practice. Australian PHC research lags behind other areas of health [2] and produces lower numbers of publications than other countries in international comparisons [3]. In addition, a better understanding of the process through which research knowledge is translated into action is required because, traditionally, research has been underutilised in PHC settings and there is a gap between the knowledge produced and its effective deployment [4].

The mechanisms through which research impacts policy and practice is sometimes referred to as a ‘pathway’ [5]. Despite the growing importance of research impact in the PHC sector, little has been published on specific pathways or the effectiveness of dissemination strategies to maximise this impact. One area of active interest is the role of intermediary organisations who serve in several roles including ‘knowledge brokers’, ‘translators of ideas’ or ‘bridging institutions’ that link researchers with users of research as a means of both exchange and translation [6, 7].

The role of intermediary organisations in increasing research impact complements the traditional individual efforts of researchers, which have primarily involved publications in academic journals and at professional conferences. The limitations of these traditional dissemination methods for achieving research impact has been questioned [8]; however, there is a dearth of information about what other dissemination strategies might be effective.

The present research was conducted as part of a larger study (The Primary Health Care Research Impact Project) designed to explore the impact of a sample of nationally-funded PHC projects. The results from this research have been reported previously in this journal [9, 10]. In this paper, we examine how CIs of primary health research projects viewed the various strategies for increasing impact and which of the available pathways they chose, and to what effect. Qualitative data describing the pathways to impact were also collected and analysed.

## Methods

### Sample frame

The sample frame consisted of 59 PHC research projects funded by the Australian Government. These projects were sponsored through either the General Practice Evaluation Program, the Primary Health Care Research Evaluation and Development Strategy, the National Health and Medical Research Council (NHMRC), or the Cooperative Research Centre for Aboriginal Health. All of these projects met the criteria of being funded after 1999 and due for completion by 2006 and having funding of more than $80,000 Australian Dollars.

### Questionnaire design

The questionnaire used in the online survey was based on an adaptation of the Buxton and Hanney Payback Framework [11]. To explore the pathways to impact, the questionnaire included items informed by the Payback Framework and by the Canadian Linkage and Exchange Model of research translation [12]. The items addressed forms of dissemination used and PHC researchers’ own assessment of the relative value of these dissemination methods (as indicated on a five-point Likert scale). Respondents were also asked to describe qualitatively what and how impacts occurred, and how important various strategies were to achieving the intended impacts.

### Administration of questionnaire

Chief Investigators (CIs) of these projects were contacted by email and provided with information about the project. Those who replied were provided with a link to an online questionnaire. A follow-up email was sent 2 weeks later and, where a telephone number was available, non-responders were contacted by phone and invited to participate in the survey.

### Analysis of data

The responses to the questions regarding methods of promoting research impact were tabulated. The perceived effectiveness of methods used, which were initially recorded on a five-point Likert scale, were subsequently coded as a dichotomous (yes/no) variable with ‘somewhat’ or ‘very relevant’ being coded as a ‘yes’. The qualitative data on impacts and how they occurred were analysed thematically using NVivo 7 and coded into broad areas of potential impact [13]. Individual quotes are included in the analysis to support the findings.

## Results

A total of 41 potential projects were identified and had investigators who could be contacted. Of these, CIs from 17 projects provided usable surveys, which are listed in Table 1. Of the 11 NHMRC funded projects, seven were Project Grants and four were Scholarships. Investigators from 14 projects could not participate as their projects were either incomplete or had failed to start, and investigators from 10 projects declined participation.

**Table 1:** Research projects included in the study

Project Title	Funding	Amount
1. A randomised controlled trial of a decision aid for prenatal screening and diagnosis	NHMRC	$269,000
2. A randomised controlled trial of physiotherapy and corticosteroid injections of lateral epicondylagia in primary care	NHMRC	$190,000
3. A rapid literature summary service to enhance evidence-based clinical decision in general practice	GPEP	$109,000
4. Audit and best practice in chronic disease	AHMRC CRCAH	$747,403
5. Cognitive screening in general practice	NHMRC	$300,000
6. Disclosure and attitudes to lesbians: outcomes in general practice (DIALOG)	NHMRC	$426,000
7. Doctors, their patients and computers: the new medical consultation - a study of the impact of computerisation	NHMRC	$103,000
8. Impact of socioeconomic disadvantage on chronic disease management in primary care: a diabetes case study	NHMRC	$258,000
9. Learning from action	CRCAH	$244,214
10. Program of resource, information and support for mothers: a community randomised trial	NHMRC	$549,000
11. Randomised controlled trial of physiotherapy injections, saline injections and exercises in the treatment of chronic low back pain	PHCRED	$97,000
12. Screening for chlamydia trachomatis with routine Pap smears in general practice: a randomised controlled trial	NHMRC	$350,000
13. Shared care for serious mental illness: caring for carers	GPEP	$93,000
14. Systematic practice-based asthma care in the Australian setting	NHMRC	$563,000
15. The evidence-based consumer: making informed decisions about menopause, hormone replacement and complementary therapies	GPEP	$97,000
16. Threats to patient safety in general practice: investigating errors in Australian primary healthcare	NHMRC	$80,000
17. Urban locational disadvantage and health: compositional and contextual determinants	NHMRC PHCRED	$608,000

*AHMRC* Aboriginal Health and Medical Research Council, *CRCAH* Cooperative Research Council for Aboriginal Health, *GPEP* General Practice Evaluation Program, *NHMRC* National Health and Medical Research Council, *PHCRED* Primary Health Care Research Evaluation and Development Strategy

### Survey of strategies used and perceived effectiveness

PHC researchers reported use of a broad range of potential dissemination strategies. The strategies reported by CIs are listed in Table 2. These strategies were divided into ‘active engagement’ (interpersonal) strategies, ‘traditional academic’ strategies and ‘non-traditional’ strategies. The most frequently used ‘active engagement’ strategies included the involvement of potential users in research design and presentations to potential users. These strategies were followed closely by the use of networks, and the dissemination of research findings to potential users. Conference presentations and publications were the most frequently used ‘traditional’ strategies. Non-traditional strategies included use of newsletters, media releases and websites.

**Table 2:** Dissemination strategies used by Chief Investigators

Method used	% Used (N)
Active engagement strategies	
Involved potential users in design of methods	82.4% (14)
Presentations to potential users	82.4% (14)
Disseminated to potential users	76.5% (13)
Use of networks	76.5% (13)
Involved potential users in developing aims	70.5% (12)
Involved potential users in interpretation of results	64.7% (11)
Traditional academic strategies	
Conference presentations	88.2% (15)
Peer-reviewed publications	76.5% (13)
Publicly available report	64.7% (11)
Non-traditional strategies	
Newsletter articles	70.5% (12)
Media releases	47.0% (8)
Project website	23.5% (4)

**Fig. 1:**
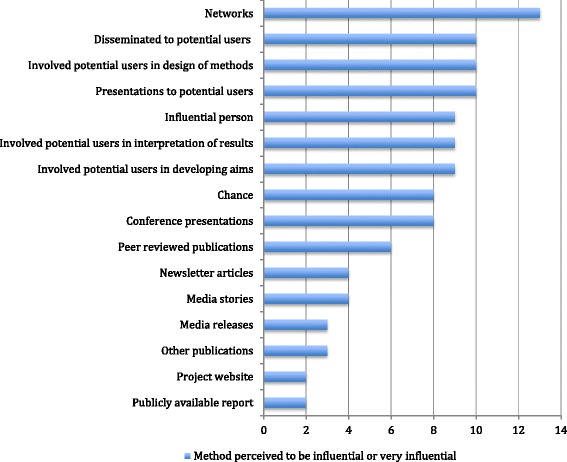
Perceived effectiveness of dissemination strategies

CIs were also asked to indicate the perceived effectiveness of these strategies (Fig. 1). Note that one or more strategies could be perceived as effective by respondents. Networks and engagement with end-users were perceived as the most effective strategies, while strategies such as newsletter articles, publicly available reports, project websites and media releases were rated substantially lower.

### Pathways to impact

In addition to the pathways proposed in the survey methodology, the responses were analysed qualitatively using thematic analysis, which identified additional pathways to impact. All pathways identified are described below.

### Active engagement strategies

#### Use of networks

Use of networks was perceived as highly influential by most CIs. Qualitative data indicated that the most effective networks were those supported by professional and other intermediary organisations (such as the Royal Australian College of General Practitioners) or knowledge exchange organisations (such as the Cooperative Research Centre for Aboriginal Health).

CIs indicated that these networks played a critical role in facilitating uptake of research findings. These networks comprised both end-users of research and people with the strategic links necessary to generate opportunities for research findings to be applied in practice. This project found that the participation of researchers in professional organisations often led to research findings contributing to professional and curriculum guidelines. For example, one respondent stated:

“*The findings have also influenced the* [professional organisation]*’s new curriculum as I was the chair of the* […] *curriculum working group. A set of guidelines for* [professionals] *and* [the minority group] *is being prepared that will provide readily accessible information about making the practice and consultation culturally sensitive to this patient group.*”

The responses indicated that PHC research was a social activity involving networks between researchers, their participants, linked organisations and universities. As one participant commented:

“*The study linked a large group of NSW GPs with a major research project at the University of Sydney. Relationships with NSW Health, the federal Department of Health and Ageing, Department of Veterans Affairs and the Royal Australian College of General Practice were developed with the university to facilitate various parts of the project.*”

Well-developed linkages between researchers, their participants, organisations and universities, however, did not guarantee that the research would be adopted. For the project quoted above, the CI reported that they did not know whether the research findings had influenced either Australian, State or local regional government policy, practice guidelines or clinical practice.

### Involvement of potential users in research

Involving potential users of research in the design and execution of the research itself has been advocated as one way of enhancing the usability of research findings. Most CIs in this study indicated that they had involved potential users in several different ways, and provided examples.

In one project, investigators involved policy-advisors as research investigators alongside senior clinicians, programme managers and other stakeholders. The state government, which contributed funding to the project, had a strong sense of ownership and was closely involved at all stages. This, as well as the project’s success in achieving health outcomes and becoming embedded in the health service management culture, appeared to contribute to the subsequent expansion of the programme.

Other projects engaged policy advisors, often through advisory committees, but were not seen as government projects. The CI of a project on asthma plans in general practice worked closely with policy advisors and completed an evaluation of a government strategy on the issue. The CI perceived that the project findings, which received timely media reports after publication, influenced the development of a funding scheme to support more comprehensive asthma consultations.

Researchers from a project on locational disadvantage held four workshops with policy advisors from state and local government, to whom preliminary results were presented. Policy advisors also sat on the project advisory committee. The researchers had little evidence of the outcome of this process at the time of participating in this study, but had been told informally that the project findings were being used to argue for increased allocations of funding for disadvantaged areas.

Four other projects in our sample involved policy advisors. Engagement ranged from being “*supportive of the intervention*”, having “*informative discussion with senior staff who had management responsibilities*”, through to formal workshops and participation on advisory committees. Involvement of one CI with multiple related government projects enhanced policy impact in that project, as did the participation of another CI on a government advisory committee. The exact manner in which the policy advisors enhanced the impact from these research projects, however, was not clear from the questionnaire responses.

### Active partnerships with clinical services

Five projects in the study achieved impact on service delivery through working closely with health services and embedding the research in the needs and concerns of these services. CIs indicated that this collaborative approach had the greatest impact on the services where the research was conducted. Together, these projects illustrate the power of action research and Continuous Quality Improvement methods to embed concepts in professional and organisational practices to the extent that they become accepted practice. In some cases, these impacts came about directly through the involvement of service managers and practitioners in the projects. In another case, the improvements in service delivery occurred through education sessions for practitioners, which meant project findings were reaching practitioners beyond those who were actually involved in the project. In another study on perinatal screening for an infectious disease, practitioners participating in a trial indicated that they were likely to incorporate the intervention in their continuing practice.

### Raising awareness of problems

Some CIs reported that they achieved impact on health service delivery by raising awareness of problems locally or more widely. This was more evident in descriptive studies where issues were identified and defined, rather than trials of specific interventions. An example was an epidemiological study which analysed existing health service data and conducted interviews, highlighting the unmet needs of a population group in an area. Following discussions with the clinical administration within the local area health service a number of changes were made to local service delivery to better meet these needs. Similarly, a project on patient safety in general practice involved a large group of GPs, policy advisors and prominent GP organisations and may have had an impact through raising awareness and changing the culture, as standards of accreditation have recently been expanded to include attention to this area. However, there was no evidence available regarding a causal link.

### Inclusion of research findings in an education programme

Research findings from at least five projects were used in health practitioner educational programmes where CIs had dual roles as educators and researchers. In several cases, participants were aware of their findings being used in other university teaching programmes or mediated through professional networks, publications, workshops or conference presentations. Including research findings in educational programmes was a pathway to broader impact on practice beyond the research participants.

In one project, an intermediary organisation funded to support research transfer provided a useful example of the benefits of prospective planning for research transfer. The investigators of this project planned to use educational programmes to increase the impact of their research and influence practice from the outset:

“*The project has specifically sought to influence educational curricula and training programs as a strategy to strengthen capacity in use of CQI* [continuous quality improvement] *concepts. This has been done through engagement of people with responsibility for such programs in the research transfer process and attracting funding for a position to specifically support this activity.*”

#### Traditional academic dissemination strategies

##### Conference presentations

Oral presentations of research findings at academic conferences are a time-honoured method of research dissemination. While less than half (47.0%) of respondents considered conference presentations as somewhat or very influential, it was the most frequent dissemination strategy used (87.2%). Participants also described other traditional dissemination platforms such as seminars and workshops. However, none of the respondents provided any specific examples of impact derived from oral presentations in any of these academic spheres.

### Presentations to professional groups

In contrast to presentations at academic meetings, respondents saw their presentations to professional groups at professional events, seminars, meetings and conferences as a more effective pathway to promote the application of their research. Some of these presentations resulted in direct impact, as in the following example:

“*The decision aid has been shown to* [practitioners] *who are requesting copies more and more. It has been used in several* [practitioners’] *study days.*”

### Peer-reviewed publications

Publications in academic journals are generally seen as the primary means of communication with the research community and the citation rate of articles is the predominant mode for assessing research impact (e.g. publishing in high impact factor journals). However, as Fig. 1 suggests, few CIs in this study perceived that their journal publications had influenced impact in terms of policy or practice. Only 35.3% of respondents indicated that peer-reviewed publications had been ‘somewhat’ or ‘very influential’ in achieving research impact. Most CIs were unable to provide evidence of uptake of research findings through peer-reviewed publications, with the exception of two projects. The outcomes of one project generated considerable debate in the literature and another was translated into Italian but no subsequent impact was reported. Three projects had their results included in systematic reviews, which could potentially lead to inclusion in professional and clinical guidelines; however, in two of the three projects the systematic review was written by the research team themselves as a side-study to the project.

### Contextual factors influencing impact

In the examples given above, the policy context in which the research was taking place influenced its possible impact. Research findings that were congruent with the current thinking of policy advisors and with current political possibilities had more chance of achieving impact. For example, a project on asthma action planning addressed a subject that was high on the agenda of both the state and federal governments. An awareness campaign was conducted in the media, and a Commonwealth Government strategy was launched at the end of the data collection phase of the study. Research findings had not led to any further policy development at the time data were collected for this research impact study, but funding had been received for an education programme and conditions looked favourable. Conversely, a project that examined health inequities produced findings that were not congruent with the direction of health policy and thus did not lead to policy change. The findings from this project did, however, receive support from individual policy officers who served on an advisory group.

One project illustrated that the impact of clinical research is also influenced by healthcare professional views. A randomised controlled trial of a medical procedure found that the intervention was not more effective than the control, yet protagonists who were teaching the technique critiqued the methods of the research and continued to advocate for the technique, with the outcome that its use was growing. In this case, the results of a single project were not able to produce definitive results on a contested issue.

The importance of community engagement is exemplified by another project, which involved a controlled trial that aimed to determine whether providing a range of services would decrease the incidence of a mental illness in a population group. It involved health practitioners and community services in several regions across many disciplines. While the trial found that the intervention was not successful in its main objective, it generated much enthusiasm among practitioners who perceived other changes as a result of the programme and continued to advocate for the intervention. This project illustrates the importance of involving community organisations and practitioners as a means to achieving research impact, but also the risks of embedding interventions into routine care before their effectiveness is proven.

## Discussion

CIs of these 17 PHC research projects identified their research projects had achieved the greatest impact through their professional and research networks, and engaging with stakeholders as part of the research development process. This was complemented by energetic dissemination of findings in many more traditional ways, including conference presentations and journal publications, but these methods were perceived as far less effective. Five prominent pathways were identified, including use of networks primarily supported via intermediary organisations, engaging policy advisors in the research, collaborations with clinical services to implement improvements, raising awareness of a problem to highlight its importance, and inclusion of results in an educational programme. CIs were unable to provide any specific information about pathways where traditional dissemination strategies, such as publication of research papers, resulted in significant impact.

The importance of networks, especially those facilitated by intermediary organisations, has not previously been noted to be of such importance in this setting. PHC research appears to be a highly interactive activity and can mobilise large networks, with many connections being formed between researchers, their participants, connecting organisations and universities. Networks were accumulated through several ways, such as the participation of CIs on committees and in professional organisations in their area of interest, collaborations between more than one university or research organisation, the formation of advisory committees which engaged policy advisors and thought leaders, and the engagement of multiple health services and whole communities in action research projects.

The role of intermediary organisations in generating impact was an unexpected finding of this study. Professional organisations sponsor conferences and seminars, facilitate the participation of practitioners as research participants, and involve researchers and others in committees to further professional curricula and guidelines. They also communicate with their networks via a range of communication strategies such as email updates and webinars and provide the structure on which networks and collaborations are built. There may be a long lead-time in developing a project, gaining support from a range of stakeholders and negotiating with collaborators. Dissemination of findings engages wider networks again with CIs presenting their findings to many different audiences. A research project provides the reason for these networks to be created and maintained, but the legacy of the research project is that the networks are there to support subsequent projects.

### Networks and track record

The reputation of CIs is built up through on-going projects. Their track record is a major factor in obtaining grants and commissions, being seen as a trusted advisor [14], being invited to join committees, and developing the personal authority that allows one to be heard and to have influence. However, the time required developing this authority is substantial and the skills in working in this setting are not necessarily those valued by academia.

Current metrics favoured by universities include research publication in high impact journals and the acquisition of peer-reviewed grants from major funders such as the NHMRC. Success on these metrics is then used as the basis for additional university research funding in Australia. NHMRC Partnerships for Better Health – Partnership Projects have recently been introduced to support new opportunities for researchers and policymakers to work together to define research questions, undertake research, interpret the findings and implement the findings into policy and practice. It is notable that the involvement of potential end-users in research featured prominently in the planning and implementation of some of the research projects surveyed.

In asking participants whether they involved potential users in their research, our approach was informed by the Canadian Linkage and Exchange Model of research transfer [12]. This model advocates for the involvement of policy advisors in all stages of the research, so as to ensure that the research meets a policy need and that those making policy decisions have a personal stake in using it. Our findings provide some examples where this has occurred, but also highlight cases where involvement has been attempted without achieving policy impact. Other findings demonstrate the potential of embedding research in the routines of health services, but again, they provide illustration of what is possible in the right circumstances rather than a standard approach for impact on service delivery.

These findings support the view that knowledge translation is a “*dynamic, interactive and multidirectional process where elements of the process can occur simultaneously or in different sequences rather than a linear or cyclical process*” [15]. Another way of saying this is that research knowledge travels through personal involvement and through the experience of jointly ‘constructing’ the knowledge gained from the research process. Knowledge constructed in this way can be described as being embedded in the social processes that gave rise to it, reflecting the interests, values and concerns of the participants. The findings are consistent with the approaches of sociology, philosophy and organisational science, which conceptualise knowledge as being created or constructed or collectively negotiated [16].

### Limitations

The reliability of this study is limited by the survey response rate and the sparse qualitative data provided in the questionnaire. Further, this study has depended not only on the memory and recall of CIs but also on the sphere of awareness of the CIs who provided the information – use of study findings in other settings may not be known to CIs, who therefore underestimate the actual impact of traditional dissemination strategies.

CIs may also exhibit recall bias and may overestimate impacts. All of the data available are self-reported and actual pathways to impact cannot be verified. Research projects assessed in this study may have gone on to achieve further impacts following completion of the survey. While a respondent may estimate the influence of a research project, evidence of a causal link between an activity or process and an outcome is more difficult to obtain.

## Conclusions

Despite the limitations described above, this study defines several pathways to research impact not previously well described in PHC research. The case studies surveyed provide insight into the pathways by which a research project may impact on its environment and suggest areas for future research. The findings of this research project have many implications for the performance and funding of PHC research. PHC researchers need to be proactive and opportunistic in the dissemination of their research findings. They need to strengthen connections with policy advisors, health service organisations, research participants, professional organisations and universities. They also need to involve policy advisors, opinion leaders, health service organisations and other potential users in their research through advisory groups or as research partners. Importantly, they need to identify the relevance of their research to policy development at all levels, including consumer groups, service providers, peak bodies, healthcare organisations and government agencies. They should also consider inclusion of their findings in educational programmes. By considering these pathways to research impact, it is likely that research findings will have a greater impact on improving policy and practice.

## Abbreviations

CI: chief investigator
NHMRC: National Health and Medical Research Council
PHC: primary healthcare
